# Intensified dose of cyclophosphamide with G-CSF support versus standard dose combined with platinum in first-line treatment of advanced ovarian cancer a randomised study from the GINECO group

**DOI:** 10.1038/sj.bjc.6604026

**Published:** 2007-10-09

**Authors:** I Ray-Coquard, D Paraiso, J-P Guastalla, B Leduc, F Guichard, C Martin, L Chauvenet, Z Haddad-Guichard, D Lepillé, H Orfeuvre, H Gautier, D Castera, É Pujade-Lauraine

**Affiliations:** 1 Centre Léon Bérard, EA 4129 sis, 28 rue Laënnec, Lyon 69008, France; 2 Centre hospitalier de l'Agglomération Montargeoise, 658 Rue des Bourgoins, Amilly 45200, France; 3 Centre hospitalier, bd du Dr Verlhac, Brive la Gaillarde 19100, France; 4 Polyclinique Bordeaux-nord, 15 rue Claude Boucher, Bordeaux 33300, France; 5 Centre hospitalier, 1 avenue de Tresum, Annecy 74000, France; 6 Hôtel-Dieu, Place du Parvis de Notre-Dame, Paris 75004, France; 7 Centre hospitalier, 7 quai Hôpital, Chalon-sur-Saône 71100, France; 8 Clinique Pasteur, 58 bd Pasteur, Evreux 27000, France; 9 Hôpital Fleyriat, 900 route de Paris, Bourg en Bresse 01000, France; 10 CMC de Bligny, 91640 Briis sous Forges, Hôpital Privé d'Antony, 1 rue Velpeau, Antony 92160, France; 11 Clinique Saint-Pierre, rue Jean Galia, Perpignan 66000, France

**Keywords:** advanced ovarian cancer, cyclophosphamide, high-dose chemotherapy, epirubicin, randomised trial

## Abstract

ICON3 trial results have suggested that CAP and carboplatin–taxol regimens as first-line treatment of advanced ovarian cancer (AOC) yield similar survival. We explored the impact of increased dose of cyclophosphamide in a modified CAP regimen on the disease-free survival (DFS) and overall survival (OS) of AOC patients. From February 1994 to June 1997, 164 patients were randomised to receive six cycles every 3 weeks of either standard CEP (S) combining cyclophosphamide (C), 500 mg m^−2^, epirubicin (E) 50 mg m^−2^, and cisplatin (P) 75 mg m^−2^ or intensive CEP (I) with E and P at the same doses, but with (C) 1800 mg m^−2^ and filgrastim 5 *μ*g kg^−1^ per day × 10 days. Response was evaluated at second-look surgery. Patient characteristics were well balanced. Except for grade 3–4 neutropaenia (S: 54%, I: 38% of cycles), Arm1 presented a significantly more important toxicity: infection requiring antibiotics, grade 3–4 thrombocytopaenia, anaemia, nausea-vomiting, diarrhoea, mucositis. Median follow-up was 84 months. DFS (15.9 *vs* 14.8 months) and OS (33 *vs* 30 months) were not significantly different between S and I (*P*>0.05). Increasing cyclophosphamide dose by more than 3 times with filgrastim support in the modified CAP regimen CEP induces more toxicity but not better efficacy in AOC.

Advanced ovarian cancer (AOC) is considered one of the most chemotherapy-sensitive epithelial malignant tumours (Ozols and Young, 1991; Trimble *et al*, 1994). First-line regimens with a platinum salt used alone or in combination (McGuire *et al*, 1996; Muggia *et al*, 2000) induce objective response in more than half of the patients. However, a majority ultimately relapse after a median interval which rarely exceeds 18 months (McGuire *et al*, 1996) suggesting the need of new therapeutic regimens.

In the 1990s, the combination of cisplatin and paclitaxel was established as first-line therapy for AOC patients after two large phase III trials had demonstrated the superiority of this combination over the then standard regimen of cyclophosphamide and cisplatin (McGuire *et al*, 1996; Piccart *et al*, 2000). A number of studies have evaluated the combination of paclitaxel and carboplatin as an alternative to the paclitaxel–cisplatin regimen (Neijt *et al*, 2000; Bookman *et al*, 2003; Ozols *et al*, 2003). They have shown that the carboplatin combination is associated with a lower incidence of non-haematologic toxicities (particularly neurotoxicity) and better quality of life, whereas no significant difference in progression-free survival (PFS) and overall survival (OS) was detected. From these findings, the International Consensus Conference in Ovarian cancer, held in Baden-Baden in 2004, concluded that carboplatin–paclitaxel was the first-line standard for AOC treatment.

This consensus however has been challenged by the results of two randomised trials comparing platinum–paclitaxel to alternative regimens. The GOG 132 study compared cisplatin alone (100 mg m^−2^), paclitaxel alone (200 mg m^−2^ over 24 h), and the combination of the two agents (paclitaxel 135 mg m^−2^ followed by cisplatin 75 mg m^−2^) in 614 women with stage III or IV disease (Muggia *et al*, 2000). There were no PFS or OS differences between the combination arm and the cisplatin-alone arm. The ICON3 trial enrolled 2074 patients with stage I through stage IV ovarian cancer who were randomised to receive carboplatin (dosed to AUC 6) in combination with paclitaxel (175 mg m^−2^ over 3 h), or a control of carboplatin (dosed to AUC 6) alone or the combination of cyclophosphamide (500 mg m^−2^), doxorubicin (50 mg m^−2^), and cisplatin (50 mg m^−2^) (CAP regimen) (ICON, 2002) no significant OS or PFS differences were between the groups. These data suggest that, at least in certain circumstances, platinum used as a single agent or in combination within a CAP regimen might be as effective as the standard paclitaxel–platinum doublet.

One approach to improve the results of chemotherapy in the first-line setting would be to optimise platinum-based regimens by increasing drug dose intensity. However, most prospective trials exploring an increase of platinum dose intensity in AOC have given negative results, suggesting that no significant benefit could be obtained with doses over 75–100 mg m^−2^ for cisplatin and AUC 5–7.5 for carboplatin (Levin *et al*, 1993).

One alternative option would be to explore the impact of increasing cyclophosphamide doses within a platinum-based regimen. The dose-limiting factor of cyclophosphamide is haematological toxicity, particularly neutropaenia. Severe neutropaenia can be improved by the administration of granulocytecolony stimulating factors (G-CSF), allowing to increase cyclophosphamide dose to a magnitude not attainable by platinum compounds because of their non-haematological or platelet dose-limiting toxicity.

These observations prompted the GINECO group to test the efficacy of chemotherapy using intensified doses of cyclophosphamide in combination with cisplatin and epirubicin against the same chemotherapy with standard doses of each drug.

## PATIENTS AND METHODS

### Study design

This study was an open, comparative, multicentric, phase III study. The primary end point was to determine the impact of increased doses of cyclophosphamide supported by filgrastim in a modified CAP regimen (CEP) on the OS of International Federation of Gynaecology and Obstetrics (FIGO) stage III–IV epithelial AOC patients. Secondary end points included evaluation of disease-free survival (DFS), patterns of recurrence, response rate, and toxicity.

### Patient selection

Eligible patients included patients with histologically documented, chemotherapy-naïve ovarian epithelial carcinoma. Other requirements included: age 18–70 years, World Health Organization (WHO) performance status ⩽2, FIGO stage III or IV, satisfactory haematological, renal, cardiac and hepatic functions, and initiation of chemotherapy within 4 weeks after initial laparotomy. Exclusion criteria were previous malignant tumour (except for treated basal cell skin cancer or *in situ* cervical cancer), history of nervous or psychiatric disorder that would preclude informed consent or compliance, infection, or severe disease including intestinal occlusion, bilirubin level >1.25 times upper normal limit, transaminases >2.5 times upper normal limit except for patients with liver metastases, white blood cell count <3.5 × 10^9^ per l, granulocyte count <2.0 × 10^9^ per l, platelet count <100 × 10^9^ per l, serum creatinine >120 *μ*mol l^−1^, symptomatic cardiomyopathy or cerebral metastasis. All patients gave written informed consent approved from regulatory authorities and ethics committees before taking part in the study.

Patients were stratified according to stage and size of the residual lesion after initial surgery: stage III and microscopic residuals, or stage III and <2 cm residuals, or stage III and >2 cm residuals, or stage IV. They were randomly assigned to receive either intensified or standard chemotherapy.

### Pre-treatment and treatment evaluation

Thoracic radiography and computed tomographic scans of the abdomen and the pelvis were performed after surgery (if realised) within 4 weeks before entry into the study. Medical history, clinical examination, performance status assessment, electrocardiogram, ventricular ejection fraction (VEF) and blood tests (complete blood cell count, serum creatinine, bilirubin, transaminases, alkaline phosphatases, and CA-125) were performed within 2 weeks. Blood cell and platelet counts were repeated weekly. Physical examination, serum CA-125 level, WHO grade toxicity assessment and blood tests were performed on day 1 of each cycle. Radiological examinations, to document the status of the disease at baseline, and serum CA-125 levels were repeated systematically at the end of the program and whenever necessary. For patients with clinical and tumour marker complete responses, treatment was followed by a second-look laparotomy.

### Treatment

#### Treatment schedule

Eligible patients were randomised to receive six cycles of either standard CEP (S) or intensive CEP (I).


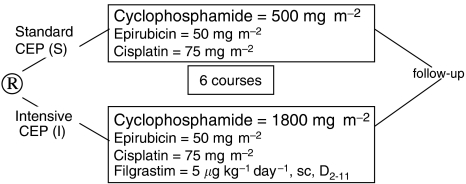


In the standard treatment arm (S) patients received at day 1 of each cycle: cyclophosphamide (ENDOXAN®) 500 mg m^−2^ administered as a 30-min intravenous infusion, followed by epirubicin (FARMORUBICINE®) 50 mg m^−2^ as a bolus infusion, and cisplatin (CISPLATYL®) 75 mg m^−2^ diluted in 500 ml of normal saline delivered as a 30-min intravenous infusion. Hydration with isotonic solution (3 l on day 1) was systematically realised for each cycle. In the intensified treatment arm (I) patient received, at day 1 of each cycle, a combination of cyclophosphamide (ENDOXAN) 1800 mg m^−2^ administered as a 30-min intravenous infusion after hyperhydration, followed by epirubicin (FARMORUBICINE) 50 mg m^−2^ and cisplatin CISPLATYL) 75 mg m^−2^. Hydration with isotonic solution (3 l on day 1) was systematically realised for each cycle, but mesna rescue was not forecasted. Filgrastim (NEUPOGEN®) 5 *μ*g kg^−1^ per day was administered from day 2 to day 11 of each cycle in the intensified arm. Filgrastim was not proposed in the standard arm, reduction of dose should be considered before.

Cycles were repeated every 3 weeks. A number of six cycles was planned, up to a maximum of nine cycles. All patients received prophylactic anti-emetic therapy with 5HT3 inhibitors.

### Toxicity and dosage modification guidelines

Toxicity was evaluated according to the WHO criteria (Perry, 1992). When patients experienced febrile neutropaenia, prolonged grade 4 neutropaenia (>7 days) or grade 4 thrombopaenia, doses of cyclophosphamide were reduced by 20% in the subsequent cycles. Doses of cisplatin were also reduced by 20% in case of grade 2 neurotoxicity. Doses of epirubicin and cyclophosphamide were also reduced by 20% in case of grade 2 stomatitis. A similar dose reduction of the three drugs was necessary in case of any grade 3 non-haematological toxicity, except digestive toxicity. Chemotherapy was stopped in case of symptomatic heart failure and creatinine serum level >150 *μ*mol l^−1^.

### Follow-up

After treatment, patients were followed every 3 months during 3 years, then every 6 months up to 5 years, and every year thereafter. Routine evaluations at each follow-up visit included physical examination and serum CA-125 level.

### Efficacy criteria

PFS was measured from the date of protocol entry to the date of first progression or death or to the date of last contact for patients who are alive and progression-free. OS was defined as the time from random assignment to death or last contact.

Clinical response was evaluated according to the WHO criteria. Those patients who were in complete clinical and serological response at the end of chemotherapy were submitted to a second-look laparotomy. Complete pathological response was defined as the absence of macroscopic and microscopic tumour lesion. Partial pathological response was defined as the persistence of microscopic lesions at second-look laparotomy in patients with residual macroscopic lesions after initial surgery, or a reduction of macroscopic lesions according to the WHO criteria.

### Statistical analysis

The study was designed to detect a difference of 15% in OS at 2 years from an expected 2-year OS of 50% in the standard arm. With a statistical power of 0.90 and a type I error of 0.05, this difference would require the inclusion of 195 patients in each arm. Statistical analysis was performed using SPSS® 9.0 software (SPSS Inc., Chicago, IL, USA 1999) and *P*-values less than 0.05 (two-sided test) were considered statistically significant. Patients who withdrew before completion of the study treatment or who were declared ineligible (*n*=9) were excluded from the primary analyses of toxicity and second look. Frequency tables and summary statistics (e.g., mean and median) were used to describe the distributions of patient characteristics and toxicity. Parametric and *χ*^2^ tests were used to test for significant differences in patient characteristics and toxicity between treatment arms. The Kaplan–Meier method was used to estimate the distributions of DFS and OS (Kaplan, 1958). Cox proportional hazards models were used to explore the associations of patient characteristics (e.g. stage and residual tumour) with patient outcome (e.g. survival) (Mantel, 1959). The score statistic was used to test for a significant difference in patient outcome on the basis of a single covariate (e.g. sex). The likelihood ratio test was used to test for the significance of a single covariate in the presence of (or adjusting for) other covariates.

## RESULTS

Enrolment began in February 1994 and was stopped in June 1996 with a lower than expected accrual of 164 patients. The main reason for this low accrual was the approval of paclitaxel in first line in the course of study enrolment. Of the 164 accrued patients, 155 were eligible and assessable, 5 had non-epithelial ovarian cancer (S (2), I (3)), 1 (I) had cardiac failure at study entry and 3 withdrew their consent before treatment (S (1), I (2)).

Patient characteristics are summarised in Table 1. The two treatment arms were well matched with respect to demographics and disease characteristics. Interestingly, like in other published series (Hoskins *et al*, 1994) less than 50% of included patients benefited of optimal debulking surgery.

### Overall and progression-free survival

**Figure 1:**
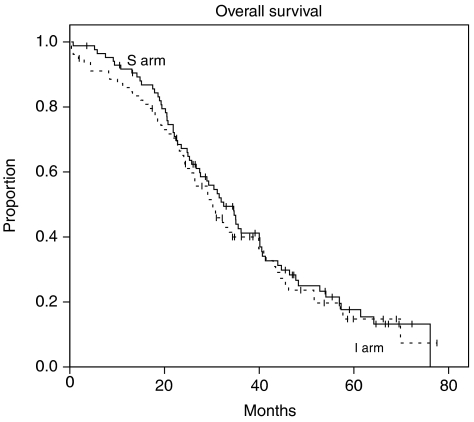
Median survival duration was 32.5 months for patients in the standard arm and 30 months for patients in the intensified arm (*P*=0.6).

**Figure 2:**
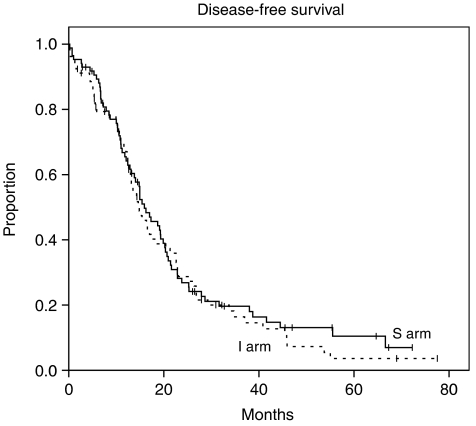
Median PFS for patients receiving the standard treatment was 15.9 months *vs* 14.8 months for patients in the intensified treatment arm (*P*=0.55).

The primary end point of the study (OS at 2 years) was 66% for the standard arm and 64% for the intensified arm (*P*=0.7). The median survival duration was 31.1 months (range 27.2–35.0) for the whole cohort. It was 32.5 months (CI 95% 27.3–37.7) for patients in the standard arm, and 30 months (CI 95% 24.7–35.7) for patients in the intensified arm (*P*=0.6) (Figure 1). Median PFS for patients receiving the standard treatment was 15.9 months (CI 95% 12.3–19.5) *vs* 14.8 months (CI 95% 11.8–17.8) for patients in the intensified treatment arm (*P*=0.55) (Figure 2).

In a univariate analysis, PFS was correlated to presence of residual lesions after initial surgery (no or microscopic lesion *vs* less than 2 cm *vs* more than 2 cm) (*P*=0.005) and to initial FIGO stage (*P*=0.05), but not to age at diagnosis, histological subtype, treatment arm, or performance status. Multivariate Cox model analysis showed that only residual lesion size after initial surgery was a significant independent prognostic factor for PFS, with a median of 33.7 months for patients without macroscopic residual lesion and 14.9 months for patients with macroscopic residual lesions (*P*=0.003).

Regarding OS, the univariate analysis identified significant prognostic values for FIGO stage (*P*=0.02), residual lesion size (*P*=0.016), and performance status (*P*=0.015), but not for age (*P*=0.34), treatment arm (*P*=0.61), or histological subtype (*P*=0.21). In a Cox model analysis, only residual lesion size after initial surgery remained statistically significantly correlated to OS (*P*=0.024) with a median survival of 46.2 months for patients without macroscopic residual lesion and 30.4 months for patients with macroscopic residual lesions.

### Pathological response

Among the 155 patients assessable for clinical and tumour marker response, only 114 (74%) had a second-look laparotomy. The reasons why second-look laparotomy was not performed in the other 41 patients are detailed in Table 2; reasons were equally distributed between the two arms.

Of the 114 patients who had a second-look laparotomy, 25 out of 63 (39%) in the standard group and 12 out of 51 (23%) in the intensified group achieved a pathological complete response (pCR) (*P*=0.46). Also, no statistical difference was noted between the two groups of patients; the intensified arm resulted in less pCR and more macroscopic residual disease. However, no information in the database provided any basis for formulating hypotheses or postulating causes, such as dose reduction, treatment delay, or patients or initial surgical characteristics.

### Toxicity

A total of 830 courses were administered (447 in S and 383 in I). The ratio of the delivered dose to the planned dose was cisplatin (S (0.99), I (0.98)), epirubicin (S (0.99), I (0.98)) and cyclophosphamide (S (0.99), I (0.94)). A total of 65 patients (78%) in the standard arm and 56 (77%) in the intensive arm received the six planned courses. No patient in either arm received more than six cycles. A dose reduction in one of the drugs was performed in 31 (15%) cycles: 20% dose reduction in 11 (5%) cycles, and 40% dose reduction in 20 (10%) cycles. In 30 out of 31 cases, dose reduction was decided because of haematotoxicity.

Chemotherapy was stopped in 34 patients, 17 in each arm. Causes of early treatment stop were as follows:

- Standard arm: toxic death [1], progressive disease [4], nausea/vomiting [1], hypersensitivity [1], elevated creatinine [1], haematological toxicity [2], others [7].
- Intensive arm: toxic death [2], progressive disease [3], cardiac arrhythmia [1], elevated creatinine [1], elevated alkaline phosphatases [1], haematological toxicity [1], fatigue [1], others [7].

Main WHO grade 3–4 toxicities are shown in Table 3. Compared to standard treatment, patients receiving intensive treatment and filgrastim support experienced a lower rate of grade 3 out of 4 neutropaenia but more frequent severe thrombopaenia and anaemia. The rate of severe infection and mucositis was significantly superior in patients receiving intensified treatment.

Grade 2 peripheral neuropathy was observed in 2 out of 82 patients of group S (2%) and in 3 out of 73 patients of group I (4%); no grade 3 was observed. Grade 2 alopecia was observed in 74 patients of group S (90%) and 69 patients of group I (95%). Grade 2 serum creatinine elevation was observed in one patient of group S (1%) and three patients of group I (4%).

## DISCUSSION

A total of 164 AOC patients were randomised to receive six cycles of CEP regimen every 3 weeks, with either standard cyclophosphamide dose (500 mg m^−2^), or intensive dose (1800 mg m^−2^) with filgrastim support.

Toxicity was higher, with more frequent severe thrombopaenia and anaemia, in the intensive than that in the standard treatment arms. Despite a lower rate of severe neutropaenia, more severe infections and mucositis were reported in patients receiving intensive cyclophosphamide. Interestingly, this increased toxicity was not associated with increased mortality and did not compromise the total drug dose or number of cycles. Thus, increasing doses of cyclophosphamide up to over 3 times, the standard dose is possible in patients with ovarian cancer within a first-line CEP regimen.

However, patients treated with the CEP regimen derived no benefit from this higher cyclophosphamide dose. PFS and OS rates were not superior for patients treated in the intensive arm compared to those in the standard arm. One of the limitations to these results is the small size of the patient sample studied. Nevertheless, it is very unlikely that a larger cohort would have allowed proving a superior efficacy of intensified cyclophosphamide dose compared to standard dose. None of the efficacy criteria tested indicated a superiority of the intensive arm over the standard. The rates of complete pathological response at second-look laparotomy, PFS and OS were all slightly inferior in patients treated with intensive cyclophosphamide. A possible explanation could be derived from the fact that the intensified arm resulted in less pCR and more macroscopic residual disease; however, this was an intriguing finding.

This study addressed two important issues for the management of first-line chemotherapy in ovarian cancer patients: the impact of high-dose therapy and the role of cyclophosphamide. The recent results of high-dose chemotherapy, including high-dose cyclophosphamide supported by peripheral blood stem cell transplantation, have yielded disappointing results in the first-line treatment of ovarian cancer. H Curé and the GINECO group have shown no benefit of high-dose carboplatin–cyclophosphamide over standard dose used as consolidation therapy in patients responding to first-line chemotherapy (Cure *et al*, 2004). Recently, Lederman *et al* (2005) reported the results of a phase III study of multi-cycle high-dose chemotherapy *vs* standard platinum-based chemotherapy in predominately optimally debulked stage IIB to IV epithelial ovarian cancer. Their data did not show a short-term benefit of the high-dose arm. There is currently no evidence of a benefit of intensive chemotherapy in ovarian cancer.

The results of the present study may contribute to the debate on the role of cyclophosphamide in the first-line setting. Before the introduction of paclitaxel in first-line, platinum combinations were considered more effective than single-agent platinum when the drug was used at the same dose (AOCT, 1991). The association of cyclophosphamide and cisplatin was adopted at that time as a standard and served as a control in the first two trials exploring the impact of paclitaxel–cisplatin combinations. However, the benefit of platinum combinations compared to single-agent platinum has long been debated; the analysis published by the Advanced Ovarian Cancer Trialists Group showed that the difference between the two regimens was at the limit of significance (ORR 0.85; CI 95%: 0.72–1.00) (AOCT, 1991). In addition, the ICON2 trial conducted in a large population of AOC patients failed to demonstrate an outcome advantage for patients treated with the CAP regimen compared to carboplatin monotherapy (ICON, 1998). The debate was revisited after the results of the four randomised trials exploring the role of paclitaxel in combination with platinum. Two of these trials used cyclophosphamide plus cisplatin as control and reported an advantage for the paclitaxel combination. In contrast, the other two trials using single-agent platinum as control did not show any benefit of the platinum–paclitaxel combination over platinum alone. From the analysis of these four trials, it has been hypothesised that a detrimental role of cyclophosphamide might explain the discordant results observed (Buyse *et al*, 2003). Our current data are not in favour of this negative impact of cyclophosphamide on patient survival for doses at least three-fold higher than standard doses, but all doses of cyclophosphamide may still impact negatively on the efficacy of cisplatin. Cisplatin dose was 75 mg m^−2^ in both arms of the study.

In conclusion, this is the only published study evaluating the dose intensity of cyclophosphamide and the two treatment arms have shown no significant difference in terms of second-look findings, complete pathological response, PFS, and OS. Our data do not support an increase of cyclophosphamide dose in a modified CAP regimen (CEP) for the first-line treatment of advanced AOC.

## Figures and Tables

**Table 1:** Patient characteristics

	**CEP**		
	**Standard (S)**	**Intensive (I)**	***P***
Patient number (%)	85 (100%)	79 (100%)	
Median age (range), in years	60 (23–70)	59 (23–70)	0.46
			
*WHO performance status*			0.66
0	24 (28%)	17 (22%)	
1	48 (56%)	50 (63%)	
2	13 (16%)	12 (15%)	
			
*FIGO stage*			0.43
III_A+B_	22 (26%)	22 (28%)	
III_C_	44 (52%)	42 (53%)	
IV	19 (22%)	15 (19%)	
Presence of ascites	31 (36%)	39 (49%)	0.15
			
*Histologic type*			0.72
Serous	59 (69%)	53 (67%)	
Endometrioid	10 (12%)	6 (8%)	
Others	16 (19%)	20 (25%)	
			
*Histologic grade*			0.81
1	19 (22%)	16 (20%)	
2	23 (27%)	22 (28%)	
3	20 (24%)	22 (28%)	
Unknown	23 (27%)	19 (24%)	
			
*Size of residual lesion after surgery*			0.77
Microscopic	12 (15%)	8 (10%)	
<2 cm	26 (30%)	25 (32%)	
⩾2 cm	47 (55%)	46 (58%)	

Abbreviation: FIGO=International Federation of Gynaecology and Obstetrics.

**Table 2:** Second-look laparotomy (SLL)

	**No. of evaluable patients (*n*=155)**	
	**Standard (S) *n*=82**	**Intensive (I) *n*=73**
*SLL (*n=*114)*	63 (77%)	51 (70%)
*Findings*		
pCR	25 (39%)	12 (23%)
Microscopic	11 (17%)	12 (24%)
<2 cm	16 (25%)	15 (29%)
>2 cm	11 (18%)	12 (24%)
		
*Not done (*n=*41)*	19 (23)	22 (30)
*Reasons*		
Stable/progressive disease	4 (21%)	3 (14%)
Early stopping due to toxicity	6 (32%)	7 (32%)
Patient refusal	1 (5%)	3 (14%)
Others	8 (42%)	9 (41%)

Abbreviation: pCR=pathological complete response.

**Table 3:** WHO grade 3 out of 4 toxicities

	**% of patients**		
	**Standard (S) *n*=82**	**Intensive (I) *n*=73**	***P***
Leucopaenia	33 (40%)	40 (55%)	0.07
Neutropaenia	53 (65%)	37 (51%)	0.07
Anaemia	17 (21%)	31 (42%)	**0.003**
Thrombocytopaenia	10 (12%)	24 (33%)	**0.001**
Nausea-vomiting	25 (30%)	24 (33%)	0.74
Diarrhoea	0	3 (4%)	0.06
Constipation	1 (1%)	3 (4%)	0.25
Mucositis	0	1 (2%)	0.28
Infections	2 (2%)	11 (15%)	**0.004**

## References

[bib1] AOCT Group (1991) Chemotherapy in advanced ovarian cancer: an overview of randomised clinical trials. Advanced Ovarian cancer trialists group. BMJ 303: 884–893 1834291 10.1136/bmj.303.6807.884 PMC1671193

[bib2] Bookman MA, Greer BE, Ozols RF (2003) Optimal therapy of advanced ovarian cancer: carboplatin and paclitaxel vs. cisplatin and paclitaxel (GOG 158) and an update on GOG0 182-ICON5. Int J Gynecol Cancer 13: 735–774 14675308 10.1111/j.1525-1438.2003.13602.x

[bib3] Buyse M, Burzykowski T, Parmar M, Torri V, Omura G, Colombo N, Williams C, Conte P, Vermorken J, International Collaborative Ovarian Neoplasm Collaborators (2003) Ovarian cancer meta-analysis project. Using the expected survival to explain differences between the results of randomized trials: a case in advanced ovarian cancer. J Clin Oncol 21: 1682–1687 12721242 10.1200/JCO.2003.04.088

[bib4] Cure H, Battista RN, Guastalla J, Fabbro M, Tubiana N, Bourgeois H, Pujade-Lauraine E (2004) Phase III randomized trial of high-dose chemotherapy (HDC) and peripheral blood stem cell (PBSC) support as consolidation in patients (pts) with advanced ovarian cancer (AOC): 5-year follow-up of a GINECO/FNCLCC/SFGM-TC study. J Clin Oncol, 2004 ASCO Annual Meeting Proceedings (Post-Meeting Edition) 22: 14S [5006]

[bib5] Hoskins WJ, McGuire WP, Brady MF, Homesley HD, Creasman WT, Berman M, Ball H, Berek JS (1994) The effect of diameter of largest residual disease on survival after primary cytoreductive surgery in patients with suboptimal residual epithelial ovarian carcinoma. Am J Obstet Gynecol 170: 974–979 8166218 10.1016/s0002-9378(94)70090-7

[bib6] International Collaborative Ovarian Neoplasm Group (1998) ICON2: randomised trial of single-agent carboplatin against three-drug combination of CAP (cyclophosphamide, doxorubicin, and cisplatin) in women with ovarian cancer. ICON collaborators. International collaborative ovarian neoplasm study. Lancet 352: 1571–1576 9843101

[bib7] International Collaborative Ovarian Neoplasm Group (2002) Paclitaxel plus carboplatin versus standard chemotherapy with either single-agent carboplatin or cyclophosphamide, doxorubicin, and cisplatin in women with ovarian cancer: the ICON3 randomised trial. Lancet 360: 505–515 12241653 10.1016/S0140-6736(02)09738-6

[bib8] Kaplan ECMP (1958) Non parametric estimation from incomplete observations. J Am Stat Assoc 53: 437–481

[bib9] Ledermann JA, Frickhofen N, Wandt H, Bengala C, Champion KM, Hinke A, Moebus V (2005) A phase III randomised trial of sequential high dose chemotherapy (HDC) with peripheral blood stem cell support or standard dose chemotherapy (SDC) for first-line treatment of ovarian cancer. J Clin Oncol, 2205 ASCO Annual Meeting Proceedings 23: 16S [5006] 10.1200/JCO.2006.09.7527 17698804

[bib10] Levin L, Simon R, Hryniuk W (1993) Importance of multiagent chemotherapy regimens in ovarian carcinoma: dose intensity analysis. J Natl Cancer Inst 85: 1732–1742 8411257 10.1093/jnci/85.21.1732

[bib11] Mantel NHW (1959) Statistical aspects of the analysis of data from retrospective studies of disease. J Natl Cancer Inst 22: 719–748 13655060

[bib12] McGuire WP, Hoskins WJ, Brady MF, Kucera PR, Partridge EE, Look KY, Clarke-Pearson DL, Davidson M (1996) Cyclophosphamide and cisplatin compared with paclitaxel and cisplatin in patients with stage III and stage IV ovarian cancer. N Engl J Med 334: 1–6 7494563 10.1056/NEJM199601043340101

[bib13] Muggia FM, Braly PS, Brady MF, Sutton G, Niemann TH, Lentz SL, Alvarez RD, Kucera PR, Small JM (2000) Phase III randomized study of cisplatin versus paclitaxel versus cisplatin and paclitaxel in patients with suboptimal stage III or IV ovarian cancer: a gynecologic oncology group study. J Clin Oncol 18: 106–115 10623700 10.1200/JCO.2000.18.1.106

[bib14] Neijt JP, Engelholm SA, Tuxen MK, Sorensen PG, Hansen M, Sessa C, de Swart CA, Hirsch FR, Lund B, van Houwelingen HC (2000) Exploratory phase III study of paclitaxel and cisplatin versus paclitaxel and carboplatin in advanced ovarian cancer. J Clin Oncol 18: 3084–3092 10963636 10.1200/JCO.2000.18.17.3084

[bib15] Ozols RF, Bundy BN, Greer BE, Fowler JM, Clarke-Pearson D, Burger RA, Mannel RS, DeGeest K, Hartenbach EM, Baergen R (2003) Phase III trial of carboplatin and paclitaxel compared with cisplatin and paclitaxel in patients with optimally resected stage III ovarian cancer: a gynecologic oncology group study. J Clin Oncol 21: 3194–3200 12860964 10.1200/JCO.2003.02.153

[bib16] Ozols RF, Young RC (1991) Chemotherapy of ovarian cancer. Semin Oncol 18: 222–232 2042062

[bib17] Perry MC (1992) Appendix-WHO toxicity guidelines. In The Chemotherapy Sources Book, Perry MC (ed) pp 1132–1144. Baltimore: Williams & Wilkins Inc

[bib18] Piccart MJ, Bertelsen K, James K, Cassidy J, Mangioni C, Simonsen E, Stuart G, Kaye S, Vergote I, Blom R, Grimshaw R, Atkinson RJ, Swenerton KD, Trope C, Nardi M, Kaern J, Tumolo S, Timmers P, Roy JA, Lhoas F, Lindvall B, Bacon M, Birt A, Andersen JE, Zee B, Paul J, Baron B, Pecorelli S (2000) Randomized intergroup trial of cisplatin-paclitaxel versus cisplatin–cyclophosphamide in women with advanced epithelial ovarian cancer: three-year results. J Natl Cancer Inst 92: 699–708 10793106 10.1093/jnci/92.9.699

[bib19] Trimble EL, Arbuck SG, McGuire WP (1994) Options for primary chemotherapy of epithelial ovarian cancer: taxanes. Gynecol Oncol 55: S114–S121 7835794 10.1006/gyno.1994.1349

